# Plasma platelet-derived growth factor receptor-β decrease correlates with blood-brain barrier damage in Alzheimer’s disease

**DOI:** 10.1186/s13024-026-00926-4

**Published:** 2026-01-13

**Authors:** Jieyin Li, Anqi Li, Chenghui Ye, Laihong Zhang, Mingxing Jiang, Guoyu Lan, Fernando Gonzalez-Ortiz, Jie Yang, Yalin Zhu, Yue Cai, Pan Sun, Lin Liu, Zhengbo He, Xin Zhou, Lili Fang, Yiying Wang, Zhen Liu, Kaj Blennow, Shaohua Ma, Xuhui Chen, Dai Shi, Tengfei Guo

**Affiliations:** 1 Institute of Neurological and Psychiatric Disorders, Shenzhen Bay Laboratory, Shenzhen, 518132 China; 2 Tsinghua Shenzhen International Graduate School (SIGS), Tsinghua University, Shenzhen, 518055 China; 3 Division of Life Science, The Hong Kong University of Science and Technology, Hong Kong, Hong Kong Special Administrative Region China; 4 Neurology Medicine Center, The Seventh Affiliated Hospital, Sun Yat-sen University, Shenzhen, 518000 China; 5 School of Biology and Biological Engineering, South China University of Technology, Guangzhou, China; 6 Shenzhen Medical Academy of Research and Translation, Shenzhen, 518132 China; 7 Institute of Neuroscience and Physiology, University of Gothenburg, Mölndal, Sweden; 8 Clinical Neurochemistry Laboratory, Sahlgrenska University Hospital, Mölndal, Sweden; 9 Department of Neurology, Xuanwu Hospital, Capital Medical University, Beijing, China; 10 Paris Brain Institute, ICM, Pitié-Salpêtrière Hospital, Sorbonne University, Paris, France; 11 Neurodegenerative Disorder Research Center, Division of Life Sciences and Medicine and Department of Neurology, Institute on Aging and Brain Disorders, University of Science and Technology of China and First Affiliated Hospital of USTC, Hefei, China; 12 Department of Neurology, Peking University Shenzhen Hospital, Shenzhen, China

**Keywords:** Plasma PDGFRβ, Qalb, BBB, Alzheimer’s disease

## Abstract

**Background:**

The blood-brain barrier (BBB) damage is frequently observed in Alzheimer’s disease (AD). However, it remains unclear whether plasma platelet-derived growth factor receptor-β (PDGFRβ) level is related to BBB damage and how it correlates with AD core pathologies, neurodegeneration, and cognitive decline.

**Methods:**

In this study, we measured cerebrospinal fluid (CSF) albumin, plasma albumin, and plasma PDGFRβ concentrations for 93 participants with paired CSF and plasma samples. We investigated the association between CSF/Plasma albumin ratio (Qalb) and plasma PDGFRβ. Subsequently, plasma PDGFRβ, phosphorylated tau (p-Tau) 217 (p-Tau217), p-Tau231, and N-terminal tau (NT1-tau) concentrations were measured in 592 participants. Of them, 519, 278, 152, 470, and 586 participants underwent testing for plasma p-Tau181, Amyloid-β (Aβ) positron emission tomography (PET), tau PET, structural magnetic resonance imaging (MRI), and MoCA, respectively. Additionally, 154 and 210 had longitudinal MRI and cognition data. We investigated the association between plasma PDGFRβ and baseline Aβ and tau PET, as well as the baseline and slope of temporal-MetaROI cortical thickness and MoCA. We did these analyses separately for the whole cohort, females, and males.

**Results:**

Plasma PDGFRβ was associated with Qalb in the whole cohort (standardized β = − 0.218 [95% confidence interval: − 0.412, − 0.024], *p* = 0.028). Mild cognitive impairment and dementia patients showed lower plasma PDGFRβ than cognitively unimpaired individuals. Lower plasma PDGFRβ levels were associated with higher Aβ and tau PET burden, thinner cortical thickness, worse MoCA scores, and a more rapid decline in cortical thickness and MoCA scores, which were particularly significant in males. Furthermore, the relationships of plasma p-Tau181, p-Tau217, p-Tau231, and NT1-tau with baseline Aβ and tau PET, as well as baseline and slopes of cortical thickness and MoCA, were much stronger in males with low (< median) plasma PDGFRβ levels compared to males with high (> median) plasma PDGFRβ levels.

**Discussion:**

This study demonstrated that decreased plasma PDGFRβ levels are associated with BBB damage, Aβ plaques, tau tangles, neurodegeneration, and cognitive decline in AD, and modulate the relationship between plasma tau biomarkers and both longitudinal neurodegeneration and cognitive decline. These findings suggest a potential plasma biomarker for detecting and monitoring BBB leakage in AD.

**Supplementary Information:**

The online version contains supplementary material available at 10.1186/s13024-026-00926-4.

## Background

The blood-brain barrier (BBB) protects brain metabolism and function, prevents harmful viruses and inflammation from the periphery, and clears potential neurotoxic compounds in the central nervous system through its complex structure, mainly composed of endothelial cells, pericytes, and astrocytes [1, 2]. The BBB function is tightly involved in Amyloid-β (Aβ) clearance [3, 4]. The pericyte around the endothelial cell in the capillary tightly controls the cerebral blood flow and maintenance of the BBB function [5, 6]. The platelet-derived growth factor receptor-β (PDGFRβ) is a membrane protein found in pericytes, which are recruited by the platelet-derived growth factor BB of endothelial cells to maintain vascular maturation and remodeling [7, 8]. The loss of pericytes leads to disruption of capillary flow and structure, especially since pericyte remodeling is slower in the aged brain [9]. Particularly, previous studies suggest that BBB damage is one of the critical pathological changes in Alzheimer’s disease (AD) [4, 10].

The BBB leakage could be reflected or measured by increases in Qalb (cerebrospinal fluid (CSF) albumin/plasma albumin ratio) [10] and CSF PDGFRβ [11–15]. Albumin can be detected in CSF because this blood protein can cross the BBB when its integrity is damaged [11, 16]. One large cohort study reported that Qalb was associated with increased BBB permeability in dementia [17]. The CSF PDGFRβ has been regarded as a biomarker of BBB dysfunction according to previous studies [11, 13–15]. Specifically, CSF PDGFRβ levels, released from the damaged pericytes, may indicate BBB dysfunction and can be detected in the early stage of AD [11, 12, 14]. CSF PDGFRβ levels have been reported to be positively related to Qalb [11, 13, 15, 16, 18], as well as with CSF fibrinogen and CSF plasminogen [11].

Emerging evidence suggests that CSF PDGFRβ levels are associated with AD pathologies [12–14] and mediate the relationship between Aβ and tau [14]. In addition to CSF BBB biomarkers, BBB damage can also be detected using a magnetic resonance imaging (MRI) water extraction technique with phase contrast arterial spin tagging [19], which has shown significant associations with AD core pathologies. Moreover, BBB dysfunction may have different mechanisms in females and males [20]. One previous extensive cohort study found that males exhibited lower BBB integrity, as reflected by Qalb, compared to females [21]. Additionally, one animal study has reported that male mice may experience more severe BBB dysfunction than females [22].

The BBB findings, based on CSF biomarkers and MRI imaging, demonstrated that BBB integrity is linked to AD pathological changes and cognitive decline, and that different sexes may exhibit distinct characteristics of BBB dysfunction in immune, endocrine, vascular, and transcriptional-mediated changes [20, 23, 24]. Two recent studies [13, 25] measured PDGFRβ concentrations in blood, but they did not investigate how serum PDGFRβ levels correlate with AD core pathologies. Particularly, it is still not fully understood whether plasma PDGFRβ concentrations can reflect BBB damage and how they relate to the Qalb, Aβ positron emission tomography (PET), tau PET, neurodegeneration, and cognitive decline in AD. Meanwhile, whether females and males exhibit different patterns in plasma PDGFRβ levels and the relationship between plasma PDGFRβ and AD pathologies and cognitive decline require further investigation.

To this end, we analyzed Chinese older adults from the Greater-Bay-Area Healthy Aging Brain Study (GHABS) [26] to reveal: (1) the correlation between plasma PDGFRβ concentrations and CSF/Plasma albumin ratio (Qalb); (2) the associations of plasma PDGFRβ with Aβ PET, tau PET, temporal-MetaROI cortical thickness, and cognitive performance; (3) whether plasma PDGFRβ levels modulate the relations of plasma p-Tau181, plasma p-Tau217, plasma p-Tau231, and plasma NT1-tau with Aβ PET, tau PET, temporal-MetaROI cortical thinning and cognitive decline as well as longitudinal changes of temporal-MetaROI cortical thinning and cognitive decline; (4) and whether those analyses above are affected by gender. Ultimately, this study aims to determine the capability of using plasma PDGFRβ to detect BBB damage and how plasma PDGFRβ concentrations relate to or modulate AD-associated pathologies and cognitive decline in AD.

## Materials and methods

### Participants

**Fig. 1:**
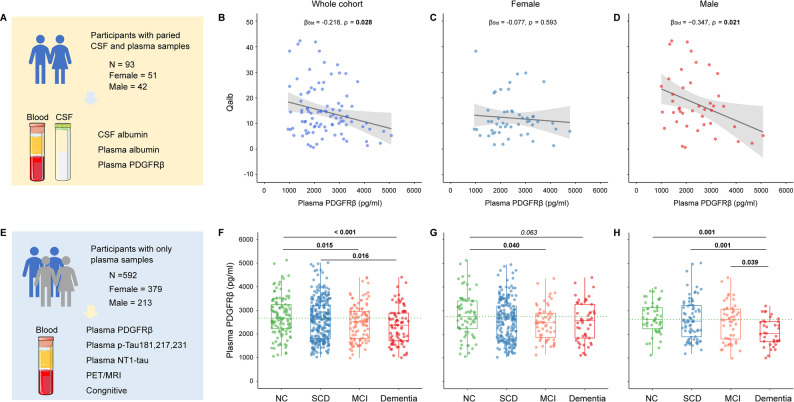
Associations of plasma platelet-derived growth factor receptor-β with Qalb and clinical diagnosis stages. (**A**) The illustration of participants with concurrent CSF and plasma samples. Association of plasma PDGFRβ with CSF/Plasma Qalb ratio in the (**B**) whole cohort, (**C**) females, and (**D**) males. (**E**) The illustration of participants with plasma samples only. Comparisons of plasma PDGFRβ in the (**F**) whole cohort, (**G**) females, and (**H**) males. Notably, the standardized regression coefficients (β_Std_) and p values of BCD were computed using a generalized linear model, adjusting for age. The standardized regression coefficients (β_Std_) and p values of FGH were computed using a generalized linear model, adjusting for age and APOE-ε4 status. CSF = cerebrospinal fluid; PDGFRβ = platelet-derived growth factor receptor-β; Qalb = CSF/plasma albumin ratio; NC = normal control; SCD = subjective cognitive decline; MCI = Mild cognitive impairment; PET = positron emission tomography; MRI = magnetic resonance imaging; p-Tau = phosphorylated tau

In this study, we included 685 participants from the GHABS cohort (clinicaltrials.gov ID: NCT06183658; Registration date: 2023-12-27) [26], which was approved by the Ethical Committees of the Shenzhen Bay Laboratory and collaborating hospitals, and was launched in May 2021. Each participant signed a written informed consent form for the GHABS project prior to enrollment. Specifically, 93 participants with concurrent CSF and plasma samples had their plasma and CSF albumin levels, as well as plasma PDGFRβ concentrations, measured (Fig. 1A). Among them, 51 were females, and 42 were males, and their median (IQR, range) ages were 67 (11, 55 ~ 86). Subsequently, we tested the concentrations of plasma phosphorylated tau (p-Tau) 217 (p-Tau217), p-Tau231, N-terminal tau (NT1-tau), and PDGFRβ among 592 participants who only had plasma samples (Fig. 1E). These participants were divided into 121 (20.4%) normal control (NC), 278 (47.0%) subjective cognitive decline (SCD), 110 (18.6%) mild cognitive impairment (MCI), and 83 (14.0%) dementia individuals following the standard protocol of the ADNI cohort [27]. Among 592 participants, 519, 278, 152, 470, and 586 underwent tests for plasma p-Tau181, Aβ PET, tau PET, structural MRI, and MoCA scores, respectively. Longitudinally, 154 and 210 GHABS participants completed at least two MRI scans and had corresponding cognitive scores. The slope of temporal-MetaROI cortical thickness (Δ Cortical thickness) and slope of MoCA (Δ MoCA) were estimated based on their longitudinal data using a linear mixed effects model (lme4 package) over time, including time, age at baseline, and sex as independent variables, and a random slope and intercept for each participant.

### Plasma biomarkers and APOE genotyping

The blood samples were collected in an EDTA tube, and the patients were fasted for one night, the day before (at least six hours). Plasma p-tau181 and p-tau217 concentrations were measured using the commercial immunoassays from Quanterix (pTau-181 V2 Advantage Kit and ALZpath pTau-217 v2). Plasma p-tau231 [28] concentrations were measured using in-house assays on the fully automated Simoa HD-X Analyzer (Quanterix Corp., Lexington, MA). Plasma PDGFRβ concentration was measured by commercial Human PDGFRβ (R&D, Catalog Number: DYC385) ELISA kits. The plasma PDGFRβ was diluted 1:20 in dilution buffer. The standard curve followed a 1:8 dilution, starting from 2000 pg/mL. The capture antibody was diluted 1:2 in PBS. 1% Sigma-Aldrich Blocker A buffer (cat#9048-46-8) in PBS was used to dilute standards and as a blocking buffer. The detection antibody was diluted 1:2. A microplate reader set to 450 nm was then used to determine the optical density. The plasma albumin (2.5 µl undiluted) was determined using a bromocresol green dye binding assay (A028-2-1) and microenzyme labeling method (Nanjing Jian Chen, China). We determined the optical density of each well after 10 min using a microplate reader set to 630 nm. APOE genotype was determined by TaqMan™ SNP (Thermo Fisher Scientific, 4351374) genotyping to define the ε2, ε3, and ε4 alleles by the two single-nucleotide polymorphisms (rs429358, rs7412) using the DNA Isolation Kit (Vazyme, DC112) based on the blood cells by centrifugation from the EDTA blood sample.

### CSF collection and measurement

Participants had to fast for one night (at least six hours), and the lumbar spinal fluid was collected the following day. Lumbar puncture was performed strictly with clinical standards, and about 2 mL of CSF was collected. The CSF sample was quickly divided and stored in a -80 °C refrigerator for subsequent analysis. The CSF albumin was measured using the Human Albumin (ALB) ELISA Kit (EHAALB, Thermo Fisher Scientific). The CSF albumin was diluted 1 to 12,000 in dilution buffer. Briefly, the standard curve starts from 1200 pg/mL. The optical density was calculated using a microplate reader set to 450 nm. The Qalb value was calculated using the following formula: (CSF albumin/plasma albumin) ×1000.

### PET and MRI imaging processing

T1 structural MRI image acquisitions were performed in a Philips Ingenia™ 3.0T MRI scanner as described previously [26]. MRI images were processed and segmented using FreeSurfer (version 7.2.0), and 68 regions of interest (ROIs) were defined according to the atlas provided in FreeSurfer. Regional gray matter volume and cortical thickness were calculated after FreeSurfer segmentation. The AD-signature temporal-MetaROI cortical thickness was calculated as the surface area-weighted average thickness of the bilateral entorhinal, fusiform, inferior temporal, and middle temporal cortex, as we described previously [29].

The acquisitions of [^18^F]-D3FSP (FSP) Aβ PET [30] and [^18^F]-flortaucipir (FTP) tau PET [31] were described in our previous study [26]. Briefly, the Aβ and tau PET images were acquired between 50 and 70 min and between 80 and 100 min post-intravenous injection of 370 MBq of [^18^F]-D3FSP and [^18^F]-flortaucipir. Composite Aβ SUVR was calculated using the average uptake of AD summary cortical regions (bilateral frontal, cingulate, parietal, and temporal regions) with the brainstem as a reference region [30]. Cortical tau tangles were evaluated by the temporal-MetaROI (entorhinal cortex, parahippocampal, amygdala, fusiform, inferior temporal, and middle temporal) FTP SUVR with the inferior cerebellar gray matter as the reference region [32].

### Statistical analysis

All statistical analyses were performed using R (version 4.4.1, R Foundation for Statistical Computing). Data were presented as median (interquartile range [IQR]) or number (%) unless otherwise noted. The normal distribution of the data in this study was determined using the Shapiro-Wilk test. The demographics and characteristics of these participants between NC, SCD, MCI, and dementia groups in this study were compared using a two-tailed Mann-Whitney U test or Fisher’s exact test at a significance level of *p* < 0.05 unless otherwise noted.

The relation between plasma PDGFRβ and Qalb values was determined using generalized linear models (GLM), controlling for age and sex. Subsequently, we compared the plasma PDGFRβ concentrations between NC, SCD, MCI, and dementia groups using GLM models, controlling for age, sex, and APOE-ε4. We did these analyses separately for the whole cohort, females, and males.

To understand the associations of plasma PDGFRβ concentrations with AD-related pathological changes and cognitive decline, we further studied the relations of plasma PDGFRβ levels with COMPOSITE FSP Aβ SUVR, temporal-MetaROI FTP tau SUVR, temporal-MetaROI cortical thickness, MoCA score, slope of temporal-MetaROI cortical thickness (Δ Cortical thickness), and slope of MoCA score (Δ MoCA) using GLM models in the whole cohort, females, and males, controlling for age, sex, and APOE-ε4.

Furthermore, we investigated how plasma PDGFRβ concentrations modulate the associations of plasma p-Tau181, plasma p-Tau217, plasma p-Tau231, and plasma NT1-Tau with COMPOSITE Aβ SUVR, temporal-MetaROI FTP SUVR, temporal-MetaROI cortical thickness, MoCA score, Δ Cortical thickness, and Δ MoCA using GLM models in females and males, controlling for the same covariates above. Notably, all the statistics in these models were obtained based on continuous variables to avoid any influence from the pre-defined thresholds. The values of plasma p-Tau181, plasma p-Tau217, plasma p-Tau231, and plasma NT1-tau were log-transformed before the following analysis to meet the normal distribution requirements. We plotted the association in individuals with high (> Median) and low (< Median) plasma PDGFRβ for illustration only. The correlation coefficient R between the outcome and predictor was calculated using the *Pearson* correlation test in individuals with high (> Median) and low (< Median) plasma PDGFRβ levels. We did these analyses separately for the whole cohort, females, and males.

## Results

### Demographics of participants

The demographic characteristics of the participants are summarized in Table 1. Among 592 participants who had plasma PDGFRβ, plasma p-tau217, p-tau231, and NT1-tau measurements, MCI and dementia patients had older ages and higher percentages of APOE-ε4 carriers than the NC and SCD groups. A higher proportion of females was found in the SCD group (201, 72.3%) compared to the other groups. Dementia patients had higher plasma p-Tau181, 217, 231, and NT1-tau levels than the NC, SCD, and MCI groups. Moreover, the MCI and dementia groups also had lower cortex thickness and MoCA scores compared to the NC and SCD groups. The longitudinal demographics of structural MRI and MoCA scores are also illustrated in Table 1. Dementia patients had more rapid rates of cortical thinning and MoCA scores decline than the NC, SCD, and MCI groups.

**Table 1:** Demographics of participants included in this study

	**NC**	**SCD**	**MCI**	**Dementia**
No., %	121, 20.4%	278, 47.0%	110, 18.6%	83, 14.0%
Age, years	66 (9.3,55–89)	67 (8.4,55–86) ^b, c^	69 (9.4,58–89) ^a, b^	71 (14.3,57–91) ^a, b^
APOE-ε4 (No., %)	31, 25.6%	61, 21.9% ^b, c^	43, 39.1% ^a, b^	37, 44.6% ^a, b^
Female (No., %)	70, 57.9%	201, 72.3% ^a, b, c^	60, 54.5% ^b^	48, 57.8% ^b^
Plasma PDGFRβ	2672.4 (1023.8)	2619.1 ^c^ (1394.4)	2519.9 ^a^ (1145.9)	2372.8 ^a, b^ (1169.9)
p-Tau217 (Log)	1.40(0.56)	1.35(0.57) ^b, c^	1.48(0.80) ^a, b, c^	2.29(0.58) ^a, b, c^
p-Tau231(Log)	2.56(0.34)	2.55(0.44) ^b, c^	2.68(0.49) ^a, b, c^	2.99(0.41) ^a, b, c^
NT1-tau (Log)	-1.35(0.49)	-1.35(0.56) ^c^	-1.31(0.55) ^a, c^	-0.89(0.57) ^a, b, c^
**Participants with p-Tau181 (*n*** **= 519)**				
No., %	106, 20.4%	247, 47.6%	95, 18.3%	71, 13.7%
pTau-181(Log)	0.51(0.43)	0.63(0.51) ^a, b, c^	0.79(0.66) ^a, b, c^	1.47(0.54) ^a, b, c^
**Participants with Aβ PET image data (*n*** **= 278)**				
No., %	47, 16.9%	138, 49.6%	55, 19.8%	38, 13.7%
Aβ PET SUVR	0.73(0.08)	0.73(0.11) ^b, c^	0.76(0.21) ^a, b, c^	1.03(0.17) ^a, b, c^
**Participants with tau PET image data (*n*** **= 152)**				
No., %	28, 18.4%	71, 46.7%	28, 18.4%	25, 16.5%
Tau PET SUVR	1.11(0.10)	1.11(0.05) ^c^	1.13(0.19) ^c^	2.02(1.08) ^a, b, c^
**Participants with MRI image data (*n*** **= 470)**				
No., %	96, 20.4%	222, 47.2%	85, 18.1%	67, 14.3%
Temporal-ROI cortical thickness	2.74 (0.15)	2.74 ^c^ (0.13)	2.73 ^c^ (0.13)	2.56 ^a, b, c^ (0.23)
**Participants with Cognitive test data (*n*** **= 586)**				
No., %	121, 20.7%	276, 47.1%	108, 18.4%	81, 13.8%
MoCA score	26(3)	26(3.25) ^b, c^	22(5) ^a, b, c^	11(6) ^a, b, c^
**Participants with longitudinal MRI image (*n*** **= 154)**				
(No., %)	27, 17.5%	81, 52.6%	28, 18.2%	18, 11.7%
Δ Temporal-ROI cortical thickness	-0.0003 (0.012)	0.0023 ^c^ (0.018)	0.0027 ^c^ (0.018)	-0.0261 ^a, b, c^ (0.021)
**Participants with longitudinal cognitive test data (*n*** **= 210)**				
(No., %)	49, 23.3%	108, 51.4%	35, 16.7%	18, 8.6%
Δ MoCA score	0.19(0.35)	0.26(0.24) ^b, c^	0.08(0.48) ^b, c^	-0.81(0.53) ^a, b, c^

Note: ^a, b, c^ indicate significant differences from the NC, SCD, and MCI groups, respectively,* p* < 0.05. Data are presented as median (interquartile range, IQR) or Number (No.) percentage (%). Data were compared using a two-tailed Mann-Whitney U test or Fisher’s exact test. Participants at baseline and longitudinal at the 2-year visit data in MRI image and MoCA scores

### Association of plasma PDGFRβ with the BBB damage factor Qalb

To determine whether plasma PDGFRβ levels can represent the BBB damage, we investigated the correlation between plasma PDGFRβ, Qalb, and CSF albumin value among 93 participants with concurrent CSF and plasma samples. The plasma PDGFRβ concentrations were negatively associated with Qalb (Fig. 1B, standardized β (β_std_) = − 0.218 [95% confidence interval (ci), − 0.412, − 0.024], *p* = 0.028) and CSF albumin (Supplementary Fig. 1A, β_std_ = − 0.236 [95% ci, − 0.426, − 0.046], *p* = 0.015). As we divided the whole cohort into females and males, significant associations between plasma PDGFRβ and Qalb and CSF albumin were observed in males (Qalb: Fig. 1D, β_std_ = − 0.347 [95% ci, − 0.641, − 0.053], *p* = 0.021; CSF albumin: Supplementary Fig. 1C, β_std_ = − 0.384 [95% ci, − 0.674, − 0.094], *p* = 0.010) but not in females (Qalb: Fig. 1C, β_std_ = − 0.077 [95% ci, − 0.359, 0.205], *p* = 0.593; CSF albumin: Supplementary Fig. 1B, β_std_ = − 0.074 [95% ci, − 0.356, 0.209], *p* = 0.610). Males had higher Qalb and CSF albumin levels than females (Supplementary Fig. 2B, D). These results support that decreased plasma PDGFRβ levels may be related to BBB permeability, particularly in males.

### Comparisons of plasma PDGFRβ between different clinical groups

To further explore how plasma PDGFRβ levels change across different clinical stages of AD, we compared the concentrations of plasma PDGFRβ between CU, SCD, MCI, and dementia groups among 592 participants with only plasma samples. Lower plasma PDGFRβ concentrations were found in MCI (β_std_ = − 0.362 [95% ci, − 0.623, − 0.101], *p* = 0.015) and Dementia (β_std_ = − 0.559 [95% ci, − 0.842, − 0.275], *p* < 0.001) patients compared to the NC individuals (Fig. 1F). Dementia patients had lower (Fig. 1F, β_std_ = − 0.365 [95% ci, − 0.616, − 0.113], *p* = 0.016) plasma PDGFRβ levels than SCD individuals. No other significant difference was found in plasma PDGFRβ concentrations. Age, sex, education, diabetes, hyperlipidemia, hypertension, and APOE-ε4 were not associated with plasma PDGFRβ concentrations (Supplementary Fig. 3–4). After dividing the whole cohort into females and males, dementia patients had lower plasma PDGFRβ levels than NC (β_std_ = − 0.776 [95% ci, − 1.199, − 0.354], *p* = 0.001), SCD (β_std_ = − 0.703 [95% ci, − 1.095, − 0.312], *p* = 0.001), and MCI (β_std_ = − 0.536 [95% ci, − 0.959, − 0.113], *p* = 0.039) groups in male individuals (Fig. 1H). In females, MCI and dementia patients had lower or marginally lower plasma PDGFRβ levels than the NC group (Fig. 1G, β_std_ = − 0.443 [95% ci, − 0.794, − 0.092], *p* = 0.040; β_std_ = − 0.422 [95% ci, − 0.806, − 0.038], *p* = 0.063).

### Associations of plasma PDGFRβ with Aβ PET, Tau PET, neurodegeneration, and cognitive decline

**Fig. 2:**
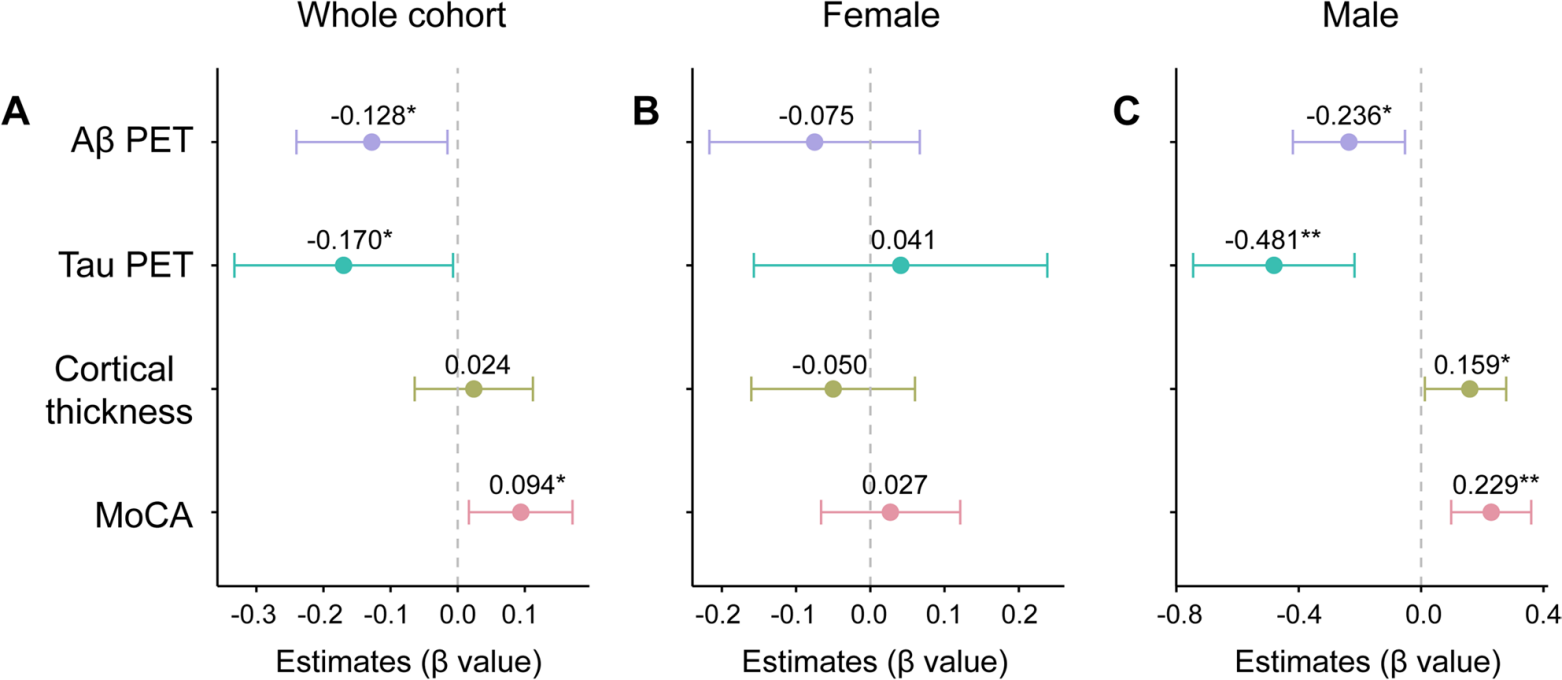
Associations of plasma platelet-derived growth factor receptor-β with Aβ PET, tau PET, cortical thinning, and cognitive decline. Association of plasma PDGFRβ with Aβ PET SUVR, tau PET SUVR, temporal-MetaROl cortical thickness, and MoCA score in the (**A**) whole cohort, (**B**) females, and (**C**) males. Notably, the standardized regression coefficients (β_Std_) and p values were computed using a generalized linear model, adjusting for age and APOE-ε4 status. Aβ = β-amyloid; PDGFRβ = platelet-derived growth factor receptor-β; PET = positron emission tomography; SUVR = standardized uptake value ratio; ROI = region of interest; MoCA = Montreal cognitive assessment

As we observed significant decreases in plasma PDGFRβ levels in MCI and dementia patients, we subsequently investigated how plasma PDGFRβ levels correlate with AD core pathologies, neurodegeneration, and cognitive decline. Plasma PDGFRβ levels were negatively associated with COMPOSITE Aβ SUVR (β_std_ = − 0.128 [95% ci: − 0.240, − 0.015], *p* = 0.026), temporal-MetaROI tau SUVR (β_std_ = − 0.170 [95% ci: − 0.333, − 0.007], *p* = 0.041), and positively related to MoCA (β_std_ = 0.094 [95% ci: 0.017, 0.171], *p* = 0.017) in the whole cohort (Fig. 2A). As we repeated these analyses in different sexes (Fig. 2B-C), lower plasma PDGFRβ levels were related to higher COMPOSITE Aβ SUVR (β_std_ = − 0.236 [95% ci: − 0.419, − 0.053], *p* = 0.023), temporal-MetaROI tau SUVR (β_std_ = − 0.481 [95% ci: − 0.745, − 0.218], *p* = 0.001), and worse temporal-MetaROI cortical thickness (β_std_ = 0.159 [95% ci: 0.012, 0.306], *p* = 0.045) and MoCA score (β_std_ = 0.229 [95% ci: 0.098, 0.360], *p* = 0.001) in males but not in females.

### Plasma PDGFRβ levels modulate the associations of plasma tau biomarkers with Aβ PET, tau PET, neurodegeneration, and cognitive decline

Subsequently, we investigated how plasma PDGFRβ levels influence the relationship between plasma biomarkers, imaging biomarkers, and cognition. Among individuals with low plasma PDGFRβ levels, much stronger positive associations was found between Aβ PET SUVR and plasma p-Tau181 (β_std_ = 0.232 [95% ci: 0.080, 0.385], *p* = 0.003), plasma p-Tau217 (β_std_ = 0.151 [95% ci: 0.014, 0.288], *p* = 0.031), plasma p-Tau231 (β_std_ = 0.213 [95% ci: 0.059, 0.366], *p* = 0.007), and plasma NT1-tau (β_std_ = 0.319 [95% ci: 0.147, 0.491], *p* < 0.001) compared to those with high plasma PDGFRβ levels in males (Table 2). In a subset of 152 participants with tau PET imaging, the positive association between plasma p-Tau181 (β_std_ = 0.541 [95% ci: 0.336, 0.745], *p* < 0.001), p-Tau217 (β_std_ = 0.462 [95% ci: 0.247, 0.677], *p* < 0.001), p-Tau231 (β_std_ = 0.381 [95% ci: 0.130, 0.631], *p* = 0.003), NT1-tau (β_std_ = 0.399 [95% ci: 0.195, 0.603], *p* < 0.001), and tau PET SUVR was more robust in individuals with low plasma PDGFRβ levels than that in individuals with high plasma PDGFRβ levels in males. In a subset of 470 participants with MRI image, we found that the negative associations of temporal-MetaROI cortical thickness with plasma p-Tau181 (β_std_ = −0.257 [95% ci: −0.400, −0.114], *p* < 0.001), plasma p-Tau217 (β_std_ = −0.184 [95% ci: −0.337, −0.032], *p* = 0.018), plasma p-Tau231 (β_std_ = −0.203 [95% ci: −0.349, −0.057], *p* = 0.007), NT1-tau (β_std_ = −0.174 [95% ci: −0.313, −0.035], *p* = 0.014) were much stronger in males with low plasma PDGFRβ levels than that in those with high plasma PDGFRβ levels. Regarding cognitive decline, higher plasma p-Tau181 (β_std_ = −0.254 [95% ci: −0.381, −0.126], *p* < 0.001), plasma p-Tau217 (β_std_ = −0.177 [95% ci: −0.310, −0.043], *p* = 0.010), plasma p-Tau231 (β_std_ = −0.213 [95% ci: −0.340, −0.087], *p* = 0.001), and plasma NT1-tau (β_std_ = −0.188 [95% ci: −0.310, −0.066], *p* = 0.003) showed stronger negative correlation with MoCA cognitive decline in males with low plasma PDGFRβ levels than that in those with high plasma PDGFRβ levels. No similar effect was found for females (Table 2).

**Table 2:** Interaction of plasma PDGFRβ and plasma tau biomarkers in predicting Aβ PET, Tau PET, neurodegeneration, and cognitive decline

	Whole cohort			Females			Males		
**Aβ PET SUVR**	β_std_	95% ci	*p*	β_std_	95% ci	*p*	β_std_	95% ci	*p*
Plasma p-Tau181	0.063	-0.042, 0.169	0.240	0.065	-0.080, 0.210	0.381	0.232	0.080, 0.385	**0.003**
Plasma p-Tau217	0.064	-0.019, 0.146	0.129	0.005	-0.109, 0.119	0.928	0.151	0.014, 0.288	**0.031**
Plasma p-Tau231	0.059	-0.041, 0.159	0.246	0.042	-0.094, 0.178	0.543	0.213	0.059, 0.366	**0.007**
Plasma NT1-tau	0.038	-0.072, 0.148	0.501	0.120	-0.021, 0.261	0.095	0.319	0.147, 0.491	< **0.001**
**Tau PET SUVR**	β_std_	95% ci	*p*	β_std_	95% ci	*p*	β_std_	95% ci	*p*
Plasma p-Tau181	0.040	-0.097, 0.177	0.569	0.183	0.025, 0.342	**0.024**	0.541	0.336, 0.745	**< 0.001**
Plasma p-Tau217	0.026	-0.110, 0.163	0.705	0.175	0.011, 0.339	**0.036**	0.462	0.247, 0.677	**< 0.001**
Plasma p-Tau231	0.033	-0.104, 0.169	0.641	0.115	-0.041, 0.271	0.147	0.381	0.130, 0.631	**0.003**
Plasma NT1-tau	0.052	-0.092, 0.196	0.479	0.132	-0.051, 0.316	0.157	0.399	0.195, 0.603	**< 0.001**
**Cortical thickness**	β_std_	95% ci	*p*	β_std_	95% ci	*p*	β_std_	95% ci	*p*
Plasma p-Tau181	-0.052	-0.147, 0.042	0.276	-0.081	-0.204, 0.042	0.196	-0.257	-0.400, -0.114	**< 0.001**
Plasma p-Tau217	-0.057	-0.143, 0.030	0.199	-0.036	-0.144, 0.072	0.514	-0.184	-0.337, -0.032	**0.018**
Plasma p-Tau231	-0.051	-0.138, 0.035	0.246	-0.058	-0.167, 0.051	0.293	-0.203	-0.349, -0.057	**0.007**
Plasma NT1-tau	-0.015	-0.105, 0.074	0.738	-0.075	-0.190, 0.041	0.207	-0.174	-0.313, -0.035	**0.014**
**MoCA**	β_std_	95% ci	*p*	β_std_	95% ci	*p*	β_std_	95% ci	*p*
Plasma p-Tau181	-0.112	-0.191, -0.032	**0.006**	-0.013	-0.115, 0.088	0.796	-0.254	-0.381, -0.126	**< 0.001**
Plasma p-Tau217	-0.065	-0.139, 0.010	0.089	-0.012	-0.103, 0.080	0.804	-0.177	-0.310, -0.043	**0.010**
Plasma p-Tau231	-0.052	-0.127, 0.023	0.172	-0.054	-0.145, 0.038	0.251	-0.213	-0.340, -0.087	**0.001**
Plasma NT1-tau	-0.034	-0.109, 0.041	0.371	-0.049	-0.142, 0.044	0.304	-0.188	-0.310, -0.066	**0.003**

Notably, the standardized regression coefficients (β_Std_) and *p* values were compared using a generalized linear model (GLM), controlling for age and APOE status (APOE-ε4 carrier or non-carrier). We analyzed the associations in individuals with high (> Median) and low (< Median) plasma PDGFRβ separately in whole, females, and males

### Associations of plasma PDGFRβ with longitudinal neurodegeneration and cognitive decline

**Fig. 3:**
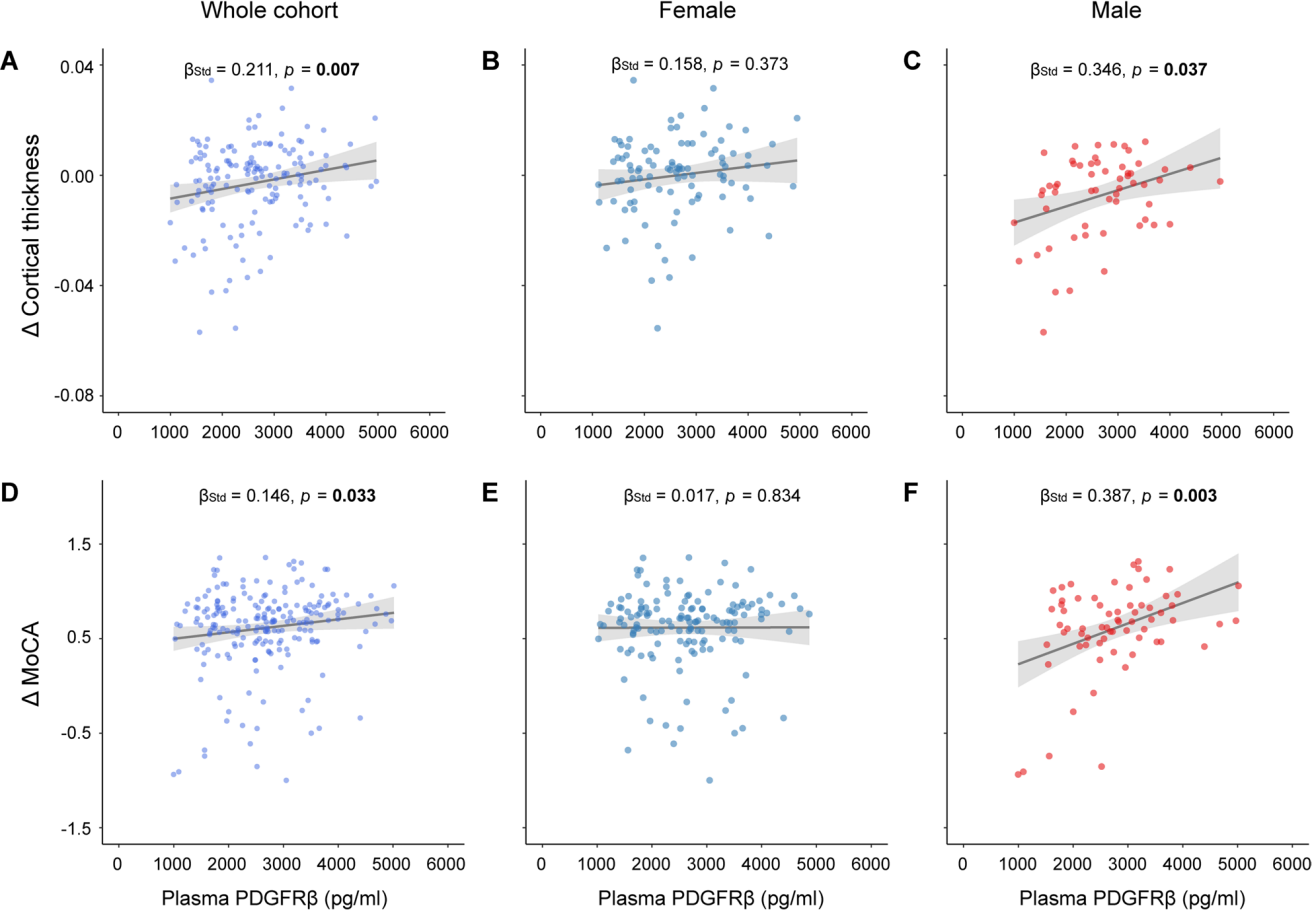
Associations of plasma platelet-derived growth factor receptor-β with longitudinal neurodegeneration and cognitive decline. Association of plasma PDGFRβ with Δ temporal-MetaROl cortical thickness in the (**A**) whole cohort, (**B**) females, and (**C**) males. Association of plasma PDGFRβ with Δ MoCA score in the (**D**) whole cohort, (**E**) females, and (**F**) males. Notably, the standardized regression coefficients (β_Std_) and p values were computed using a generalized linear model, adjusting for age and APOE-ε4 status. PDGFRβ = platelet-derived growth factor receptor-β; ROI = region of interest; MoCA = Montreal cognitive assessment

Longitudinally, we found that lower plasma PDGFRβ levels were related to faster rates of decrease in temporal-MetaROI cortical thickness (β_std_ = 0.211 [95% ci: 0.059, 0.364], *p* = 0.007), and MoCA scores (β_std_ = 0.146 [95% ci: 0.012, 0.280], *p* = 0.033) (Fig. 3A, D). As we repeated these analyses in different sexes, lower plasma PDGFRβ levels were associated with faster rates of temporal-MetaROI cortical thinning (β_std_ = 0.346 [95% ci: 0.085, 0.606], *p* = 0.037) and cognitive decline (β_std_ = 0.387 [95% ci: 0.162, 0.613], *p* = 0.003) in males (Fig. 3C, F), not in females (Fig. 3B, E).

### Plasma PDGFRβ levels modulate the associations of plasma tau biomarkers with longitudinal neurodegeneration and cognitive decline

**Fig. 4:**
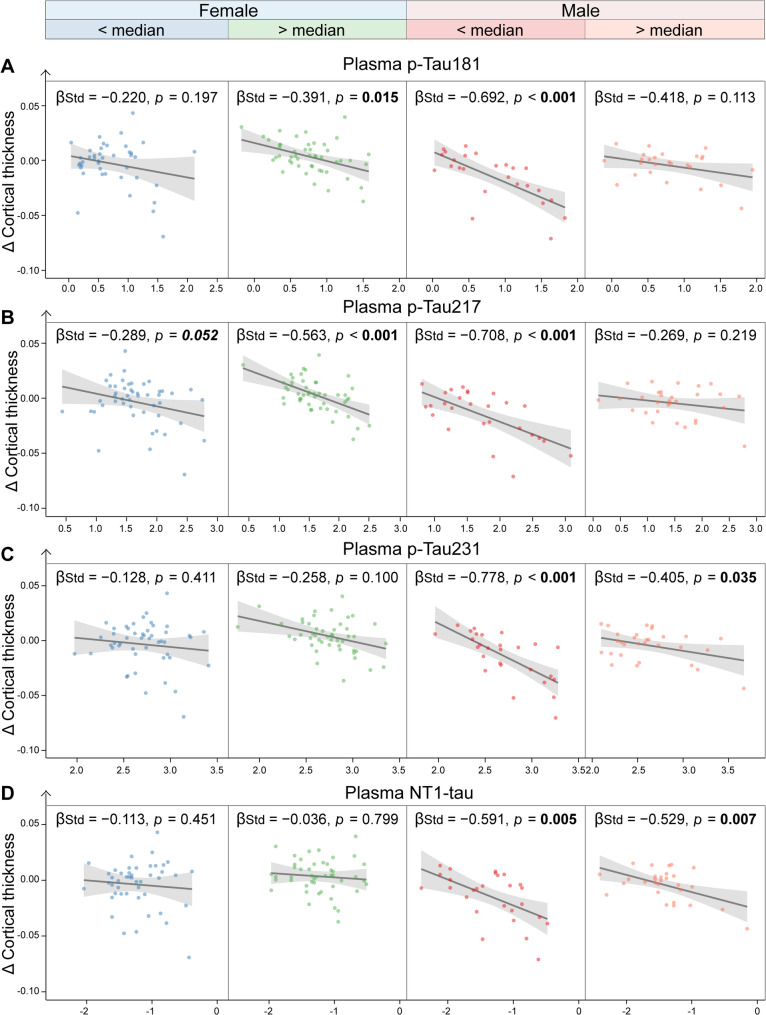
Interaction of plasma platelet-derived growth factor receptor-β and plasma tau biomarkers with longitudinal cortical thinning. The illustration of the association of plasma p-tau181 (**A**), p-tau217 (**B**), p-tau231 (**C**), and NT1-tau (**D**) with Δ temporal-MetaROI cortical thickness in different subgroups (female < Median, and female > Median, male < Median, and male > Median) of plasma PDGFRβ. The points and solid lines represent the participants in each subgroup, along with the regression lines (95% confidence interval). PDGFRβ = platelet-derived growth factor receptor-β; ROI = region of interest; p-Tau = phosphorylated tau; NT1-tau = N-terminal tau

**Fig. 5:**
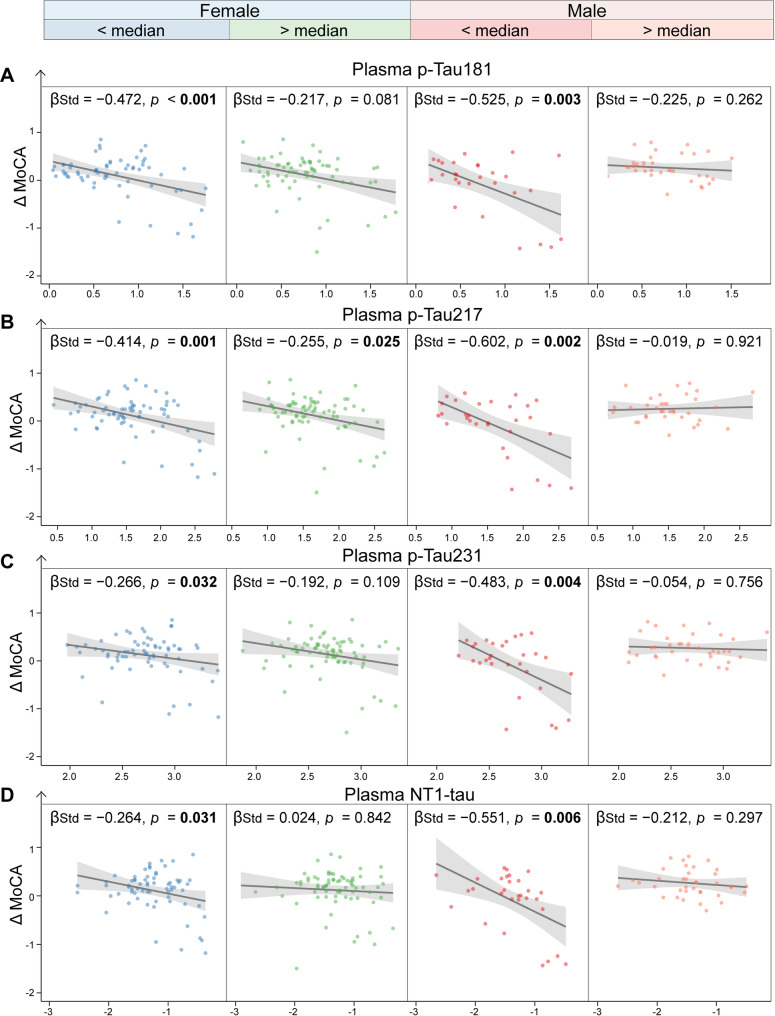
Interaction of plasma platelet-derived growth factor receptor-β and plasma tau biomarkers with longitudinal cognitive decline. The illustration of the association of plasma p-tau181 (**A**), p-tau217 (**B**), p-tau231 (**C**), and NT1-tau (**D**) with Δ MoCA score in different subgroups (female < Median, and female > Median, male < Median, and male > Median) of plasma PDGFRβ. The points and solid lines represent the participants in each subgroup, along with the regression lines (95% confidence interval). PDGFRβ = platelet-derived growth factor receptor-β; p-Tau = phosphorylated tau; NT1-tau = N-terminal tau; MoCA = Montreal cognitive assessment

All plasma tau biomarkers were negatively associated with more rapid rates of temporal-MetaROI cortical thinning and decline in MoCA scores in the whole cohort, particularly in males (Supplementary Fig. 5–6). The negative associations of Δ Cortical thickness with plasma p-Tau181 (β_std_ = − 0.692 [95% ci: − 1.010, − 0.374], *p* < 0.001), plasma p-Tau217 (β_std_ = − 0.708 [95% ci: − 1.047, − 0.369], *p* < 0.001), plasma p-Tau231 (β_std_ = − 0.778 [95% ci: − 1.106, − 0.451], *p* < 0.001), NT1-tau (β_std_ = − 0.591 [95% ci: − 1.004, − 0.178], *p* = 0.005) were more robust in males with low (< median) plasma PDGFRβ levels than males with high (> median) plasma PDGFRβ levels, females with low (< median) and high (> median) plasma PDGFRβ levels (Fig. 4). Likewise, much stronger negative association was found between Δ MoCA and plasma p-Tau181 (β_std_ = − 0.525 [95% ci: − 0.877, − 0.174], *p* = 0.003), plasma p-Tau217 (β_std_ = − 0.602 [95% ci: − 0.978, − 0.227], *p* = 0.002), plasma p-Tau231 (β_std_ = − 0.483 [95% ci: − 0.809, − 0.157], *p* = 0.004), plasma NT1-tau (β_std_ = − 0.551 [95% ci: − 0.943, − 0.158], *p* = 0.006) in males with low (< median) plasma PDGFRβ levels compared to other groups (Fig. 5).

## Discussion

In this study, we investigated plasma PDGFRβ levels and their association with CSF/plasma albumin ratio (Qalb), Aβ PET, tau PET, cortical thinning, and cognitive decline in a large Chinese aging population. We found that plasma PDGFRβ levels were negatively associated with the CSF/plasma albumin ratio and showed significant decreases in MCI and dementia patients compared to NC individuals. Lower plasma PDGFRβ concentrations were associated with higher Aβ plaques and tau tangles, greater cortical thinning, poorer cognitive function, and faster rates of cortical thinning and cognitive decline in the entire cohort. These associations were significant, particularly in males. Furthermore, more robust associations of plasma p-Tau181, plasma p-Tau217, plasma p-Tau231, and plasma NT1-tau with Aβ plaques, tau tangles, AD-signature cortical thinning, worse cognitive function, more rapid rates of cortical thinning, and cognitive function were found among individuals with low plasma PDGFRβ levels compared to those with high levels in males. Together, these findings demonstrated that lower levels of plasma PDGFRβ correlate with BBB leakage, cortical Aβ and tau aggregation, AD-signatured cortical thinning, and cognitive decline, and moderate the relationships between peripheral and cerebral AD pathologies in AD (particularly in males), providing an effective plasma BBB biomarker to detect and monitor BBB dysfunction and predict the progression of AD.

The plasma PDGFRβ concentrations were measured for the first time in a large dataset of aging individuals. The CSF/plasma albumin ratio has been regarded as a standard for quantifying BBB leakage in neurodegenerative diseases and is commonly used to evaluate the performance of fluid or imaging biomarkers for detecting BBB damage [17, 19, 33, 34]. In this study, we found that plasma PDGFRβ levels negatively correlate with the CSF/plasma albumin ratio, suggesting that lower plasma PDGFRβ levels are associated with greater BBB leakage in AD. Increased CSF PDGFRβ levels have been observed in patients with cognitive impairment, as reported in previous studies [12–14]. Additionally, BBB imaging indicators derived from MRI techniques also showed significant changes in AD patients [16, 19]. Unlike CSF PDGFRβ, we found that plasma PDGFRβ levels were decreased in MCI and dementia patients compared to NC individuals. Lower plasma PDGFRβ levels were associated with higher Aβ plaque and tau tangle, as well as worse cognitive function, in older adults, particularly in males.

In line with our findings, biomarker studies [13, 14, 16] have also demonstrated that CSF PDGFRβ levels are significantly associated with AD pathologies, although no sex effect was found [13]. Besides, imaging studies [19, 35] also observed a significant correlation between BBB dysfunction and AD pathologies. Together with these reports, MCI or AD patients may exhibit BBB dysfunction compared to healthy individuals, and these BBB damages can be measured by the CSF/plasma albumin ratio and PDGFRβ, as well as dynamic contrast-enhanced or diffusion-prepared arterial spin labeling MRI imaging. Notably, these biomarkers may reflect different aspects of damage to BBB integrity. In the present study, we further demonstrated that reduced plasma PDGFRβ concentrations may indicate damage to BBB integrity in AD, particularly in males. This blood-based biomarker may offer a more convenient tool for detecting BBB integrity in AD patients.

Another primary finding of this study was that the relationships of plasma p-Tau181, p-Tau217, p-Tau231, and NT1-tau with Aβ-PET, tau-PET, temporal-MetaROI cortical thickness, and MoCA cognitive score, as well as longitudinal temporal-MetaROI cortical thinning and cognitive decline, were much stronger in males with low (< median) plasma PDGFRβ levels compared to males with high (> median) plasma PDGFRβ levels. Consistent with our findings, Wang and colleagues [14] also noticed a positive relationship between CSF Aβ42 and CSF p-Tau181 in participants with high CSF PDGFRβ levels (indicating greater BBB damage) compared to those with low levels. Our lab [28, 36] and other groups [37–42] have demonstrated the predictive effect of plasma p-Tau and NT1-tau in AD pathologies and cognitive decline. The present study’s findings suggest that individuals with more BBB damage tend to have stronger relationships between AD pathologies in the peripheral system (e.g., plasma p-Tau) and those in the central nervous system (as observed in brain imaging) in older adults. The combination of plasma PDGFRβ and plasma p-Tau biomarkers may improve the power to predict cortical Aβ plaque and tau tangle aggregation, as well as neurodegeneration and cognitive decline in AD.

In line with our findings that plasma PDGFRβ-associated BBB damage was primarily effective in males, previous studies have observed sex differences in BBB dysfunction [43]. Additionally, BBB integrity may be influenced by sex hormones in the development and progression of neurodegenerative diseases [20]. Supporting this, a DCE-MRI BBB study reported that males may have worse BBB integrity than females in the cingulate and occipital cortices [23]. A new non-invasive diffusion-prepared pseudo-continuous arterial spin labeling MRI technique has been reported to reveal more age-related BBB damage in the parietal and temporal regions compared to females [44]. Recently, a proteomic study demonstrated that males exhibit more BBB dysfunction compared to females [45]. Converging evidence from animal models further indicates that male mice have lower levels of BBB tight junction proteins and are influenced by factors such as ethanol exposure in adolescents [46] and lipopolysaccharide exposure [47], with females typically experiencing a lesser impact than males. It is important to note that plasma PDGFRβ levels did not differ significantly between females and males in our analyses, and neither did previous studies [11–14] observe a significant sex effect in CSF PDGFRβ levels. However, plasma PDGFRβ levels showed different associations with CSF/plasma albumin ratio and AD-related pathologies in both the CSF/Plasma sub-cohort and the plasma-only sub-cohort. Together, it is likely that males may experience more severe BBB leakage issues or detrimental downstream effects related to BBB damage compared to females. Future investigation is required to elucidate the potential mechanism underlying this sex effect on plasma PDGFRβ.

In this study, we analyzed PDGFRβ concentrations in plasma, as well as the CSF-to-plasma albumin ratio. We demonstrated their negative correlation in older adults, particularly in males. Furthermore, we comprehensively investigated the relationship between reduced levels of plasma PDGFRβ and cortical Aβ plaque, tau tangle, AD-signature cortical thinning, and cognitive decline in a large dataset. Meanwhile, we observed more robust positive associations between plasma p-Tau and cortical Aβ and tau aggregation, as well as more pronounced negative plasma p-Tau-related AD signatures of cortical thinning and cognitive decline, among male individuals with lower plasma PDGFRβ levels. These findings provide a critical reference for using plasma PDGFRβ to detect BBB damage in older adults. However, this study has several limitations. First, we currently have limited concurrent blood and CSF samples in our cohort, which restricts our ability to explore the relationship between plasma PDGFRβ and serum PDGFRβ, as well as their correlation with CSF PDGFRβ. The decreased plasma PDGFRβ levels correlating with BBB damage may be specific only to the PDGFRβ kit used in this study. Future investigations using paired plasma, serum, and CSF samples, along with PDGFRβ levels measured by different kits or proteomic analyses, would help clarify PDGFRβ levels in plasma, serum, and CSF in AD. Second, the GHABS participants were highly selected from the community, which may not accurately represent the broader aging population in the real world. Further studies with other community cohorts will enable exploration of how plasma PDGFRβ concentrations vary across different clinical stages of AD and by sex. Third, longitudinal data on plasma and imaging biomarkers are currently unavailable. Thus, we still need more longitudinal data to determine how plasma PDGFRβ levels change over time in the future.

## Conclusions

In summary, we measured plasma levels of PDGFRβ and CSF/plasma albumin ratio in a well-established AD cohort with multiple fluid and imaging biomarkers. We observed a negative correlation between plasma PDGFRβ levels and the CSF/plasma albumin ratio, indicating that the low plasma PDGFRβ levels may represent damage to the BBB integrity in AD. Furthermore, we found a significantly stronger association between plasma p-Tau181, p-Tau217, p-Tau231, and NT1-tau and brain imaging (Aβ PET, tau-PET, temporal-MetaROI cortical thickness) as well as cognitive function among individuals with low plasma PDGFRβ levels compared to those with high plasma PDGFRβ levels. These findings suggest that a combination of plasma p-Tau and PDGFRβ would enable us to predict pathological changes in the AD brain more accurately. For the first time, this study demonstrated that decreased plasma PDGFRβ levels are associated with BBB damage, providing a potential plasma biomarker for detecting and monitoring BBB leakage in AD, particularly in males. However, more investigation is still required to determine its capability of detecting BBB damage in females.

## Supplementary Information

Below is the link to the electronic supplementary material.

Supplementary Material 1

## Data Availability

The data used in the current study were obtained from the GHABS cohort. Derived data is available from the corresponding author on request by any qualified investigator, subject to a data use agreement.

## Abbreviations

AD: Alzheimer’s disease
APOE: Apolipoprotein E
Aβ: β-amyloid
BBB: Blood-brain barrier
CSF: Cerebrospinal fluid
MCI: Mild cognitive impairment
MoCA: Montreal cognitive assessment
MRI: Magnetic resonance imaging
NC: Normal control
NT1-tau: N-terminal tau
PDGFRβ: Platelet-derived growth factor receptor-β
PET: Positron emission tomography
p-Tau: Phosphorylated tau
Qalb: CSF/plasma albumin ratio
ROI: Region of interest
SCD: Subjective cognitive decline
SUVR: Standardized uptake value ratio
