# Supplementary material for: Plasma platelet-derived growth factor receptor-β decrease correlates with blood-brain barrier damage in Alzheimer’s disease
Source: Mol Neurodegener. 2026 Jan 13;21:12. doi: 10.1186/s13024-026-00926-4 (PMC12888681; doi:10.1186/s13024-026-00926-4)
Supplement: Supplementary file 1 — Supplementary Material 1 [file 13024_2026_926_MOESM1_ESM.pdf]

Supplementary Material

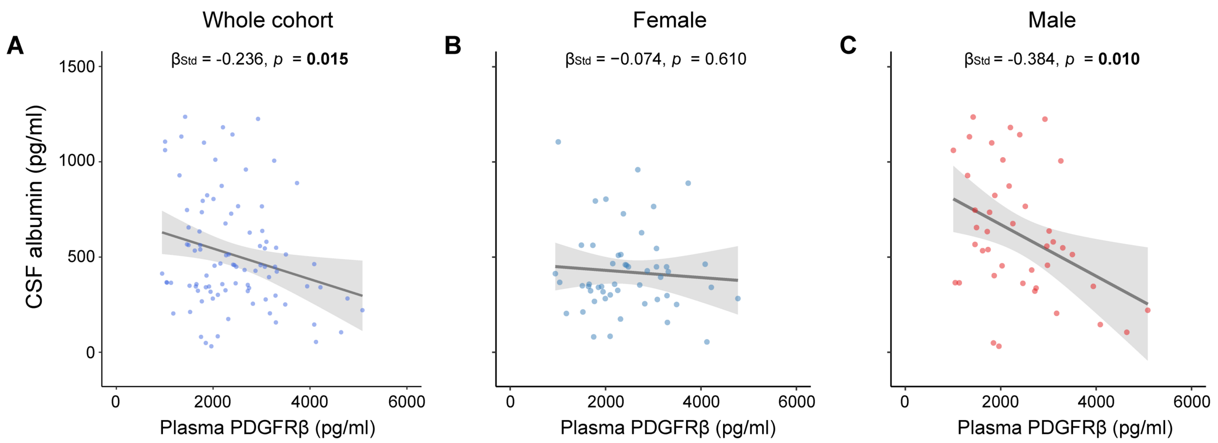

Supplementary Figure 1. Associations of plasma PDGFRβ with CSF albumin in the (A) whole cohort, (B) females, and (C) males. Notably, the standardized regression coefficients (β_{Std}) and values were computed using a generalized linear model, adjusting for age. CSF = cerebrospinal fluid; PDGFRβ = platelet-derived growth factor receptor-β.

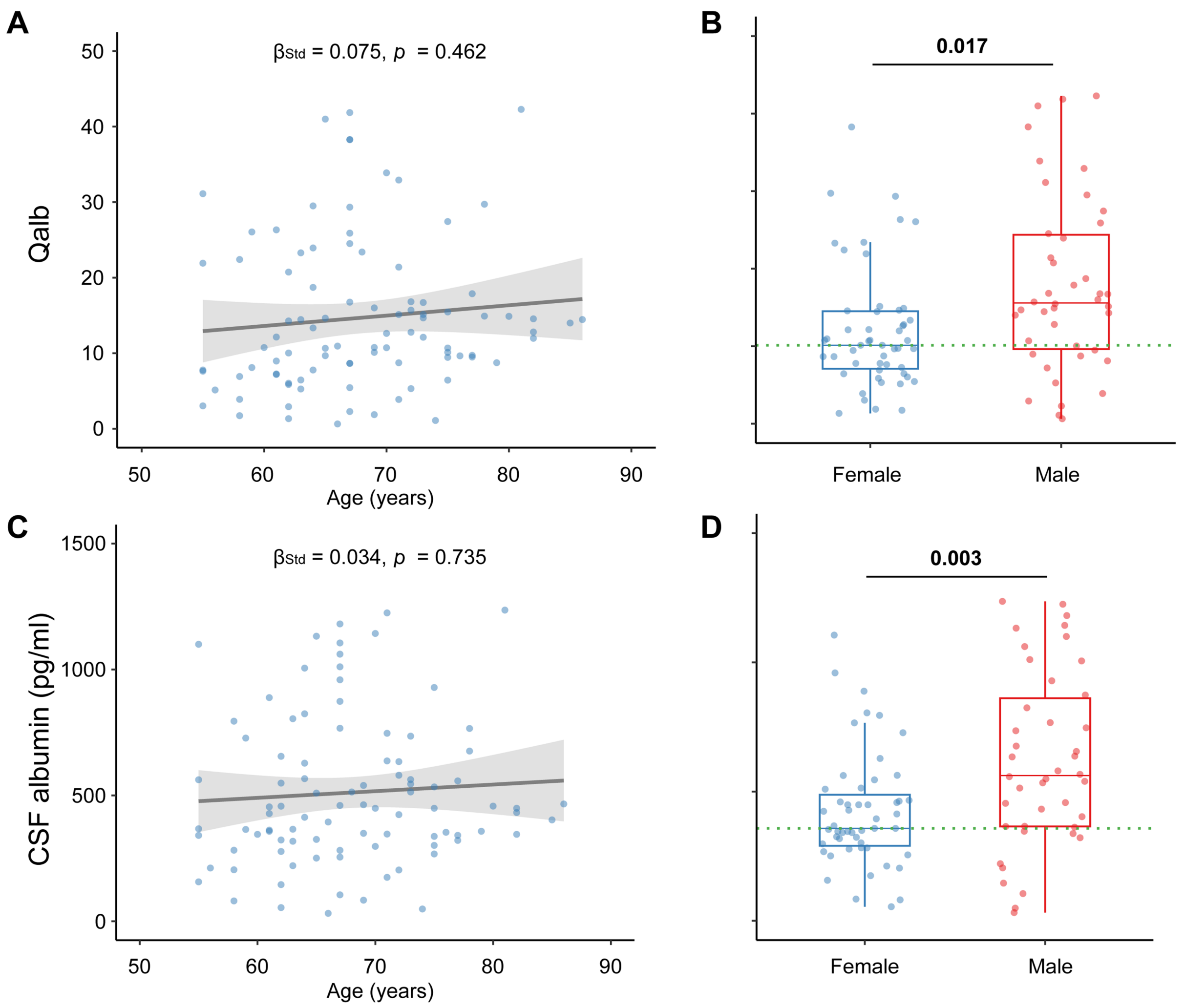

Supplementary Figure 2. Associations of plasma platelet-derived growth factor receptor-β with Qalb and CSF albumin. (A) Association of Qalb with age. (B) Comparison of Qalb between females and males. (C) Association of CSF albumin with age. (D) Comparison of CSF albumin between females and males. Notably, the standardized regression coefficients (β_{Std}) and p values were computed using a generalized linear model, adjusting for sex. CSF = cerebrospinal fluid; Qalb = CSF/plasma albumin ratio.

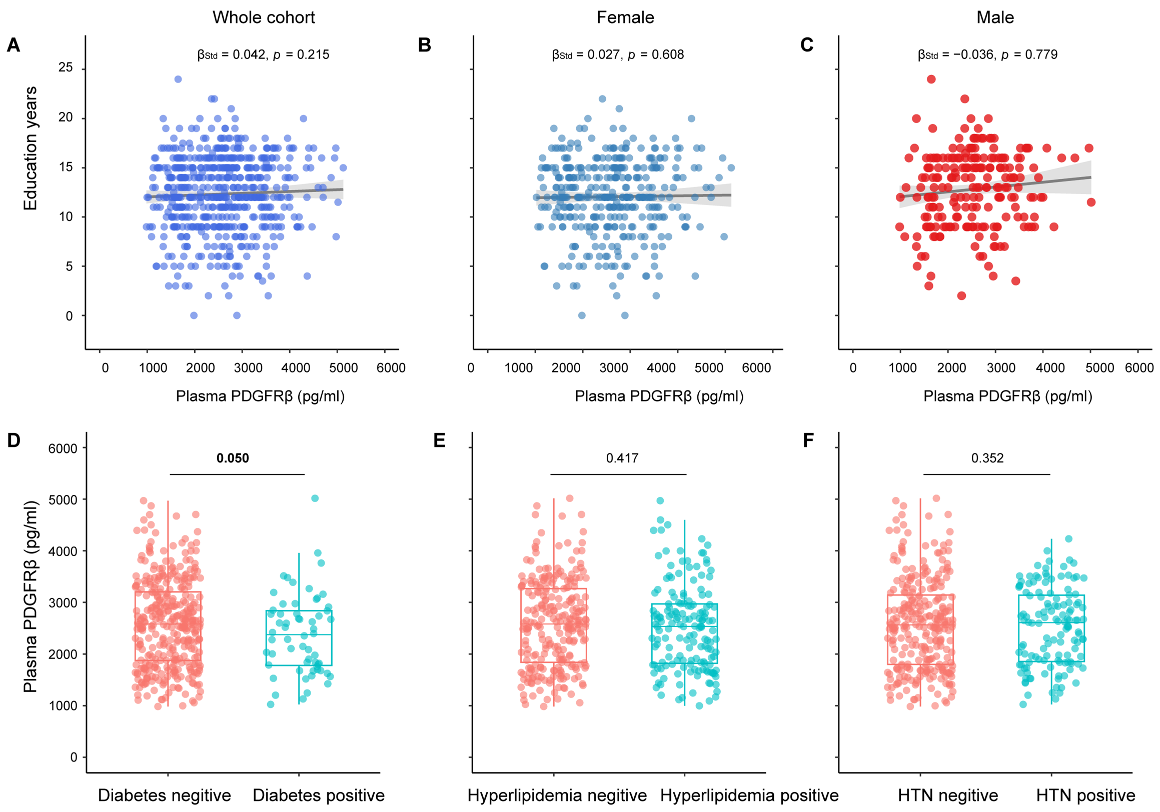

Supplementary Figure 3. Association of plasma PDGFRβ with education years in the whole cohort (A), females (B), and males (C). Comparisons of plasma PDGFRβ in the diabetes (D), dyslipidemia (E), and hypertension (F).

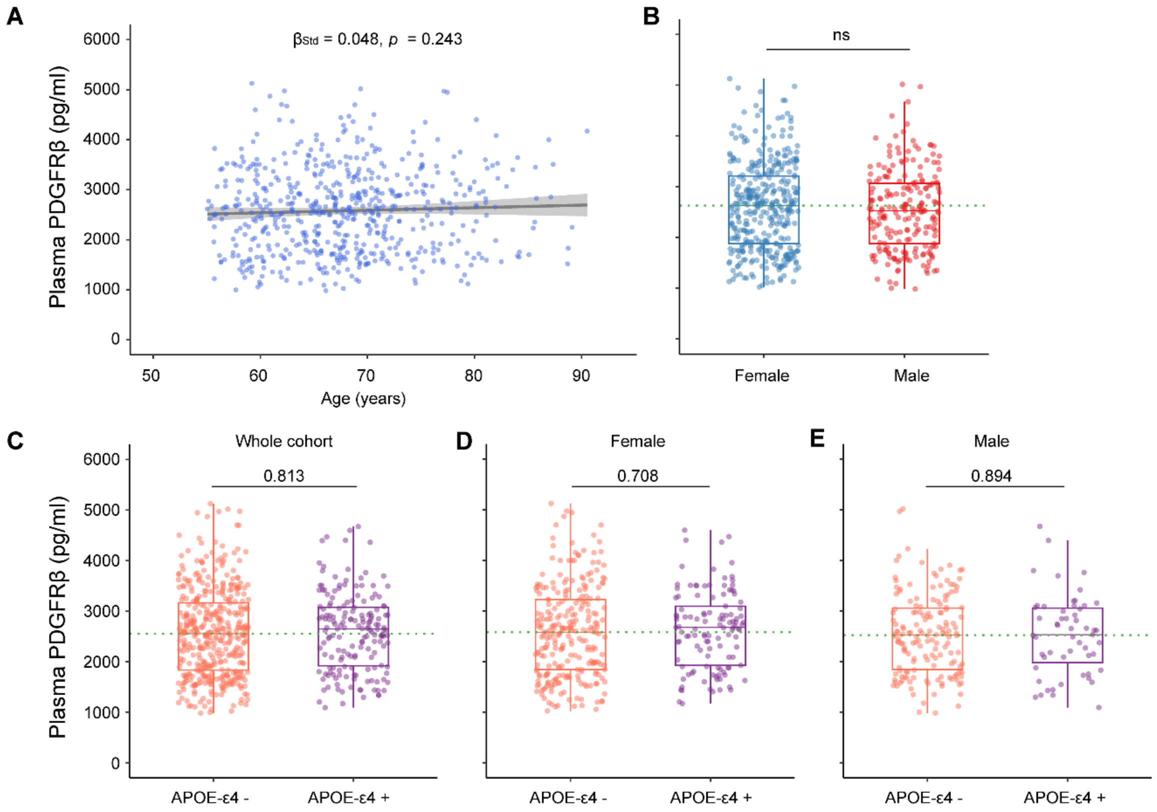

Supplementary Figure 4. Association of plasma platelet-derived growth factor receptor-β with age, sex, and APOE-ε4. (A) Association of plasma PDGFRβ with age. (B) Comparisons of plasma PDGFRβ between females and males. (C-E) Comparisons of plasma PDGFRβ between APOE-ε4 carriers and non-carriers in the whole, female, and male cohorts. Notably, the standardized regression coefficients (β_{Std}) and p values were computed using a generalized linear model, adjusting for sex and APOE-ε4 status. The p-values for the comparisons are shown at the top, adjusted for age, sex, or APOE-ε4 status. PDGFRβ = platelet-derived growth factor receptor-β; APOE ε4-, APOE-ε4 no-carrier; APOE ε4+, APOE-ε4 carrier.

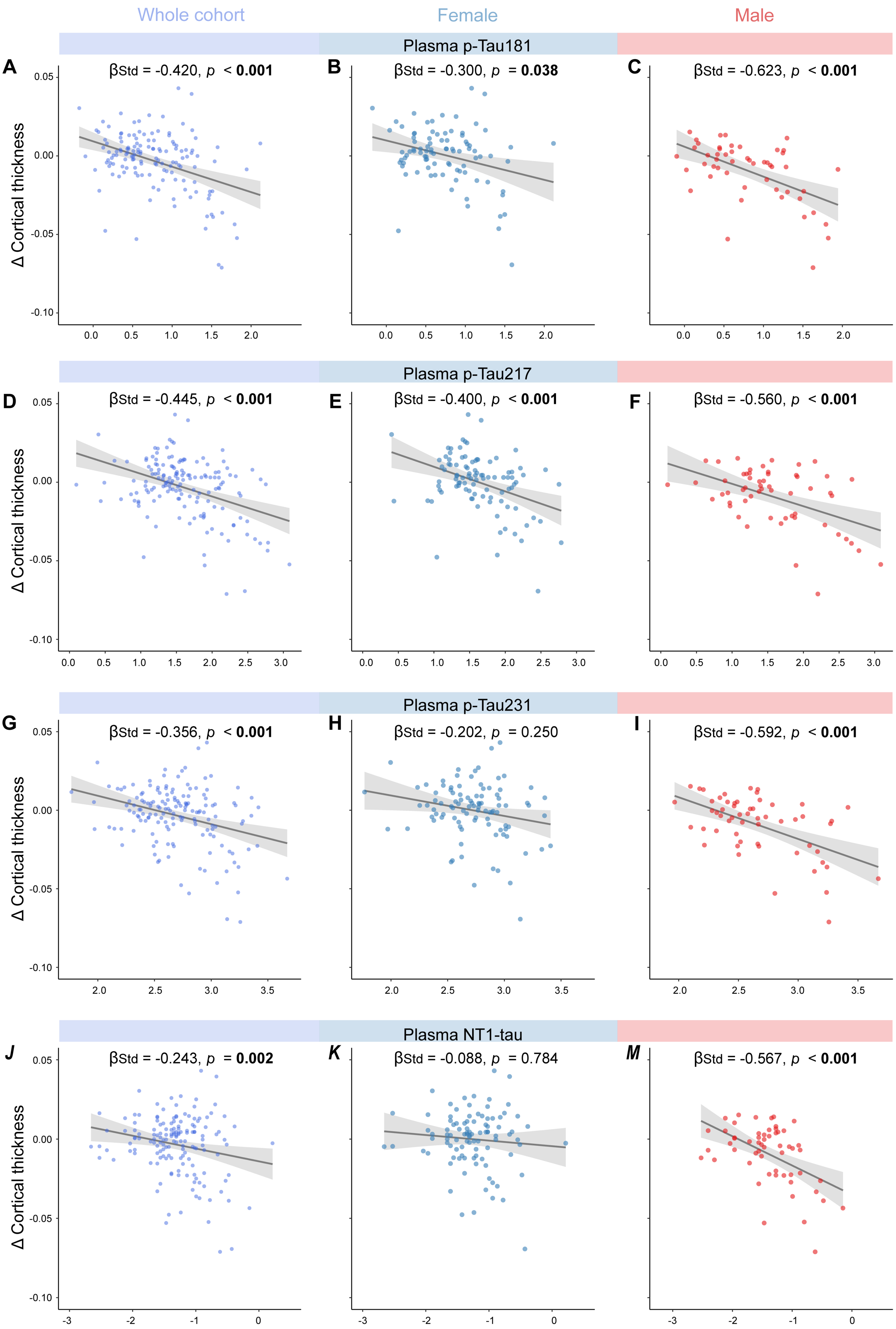
Whole cohort
Female
Male
Plasma p-Tau181
A βStd = -0.420, p < 0.001
B βStd = -0.300, p = 0.038
C βStd = -0.623, p < 0.001
Plasma p-Tau217
D βStd = -0.445, p < 0.001
E βStd = -0.400, p < 0.001
F βStd = -0.560, p < 0.001
Plasma p-Tau231
G βStd = -0.356, p < 0.001
H βStd = -0.202, p = 0.250
I βStd = -0.592, p < 0.001
Plasma NT1-tau
J βStd = -0.243, p = 0.002
K βStd = -0.088, p = 0.784
M βStd = -0.567, p < 0.001
Δ Cortical thickness

Supplementary Figure 5. Associations of longitudinal cortical thinning with plasma tau biomarkers. Association of Δ temporal-MetaROI cortical thickness with plasma p-tau181 (A-C), p-tau217 (D-F), p-tau231 (G-I), and NT1-tau (J-M) in different subgroups (whole, female, male). Notably, the standardized regression coefficients (β_{Std}) and values were computed using a generalized linear model, adjusting for age and APOE-ε4. p-Tau = phosphorylated tau; NT1-tau = N-terminal tau.

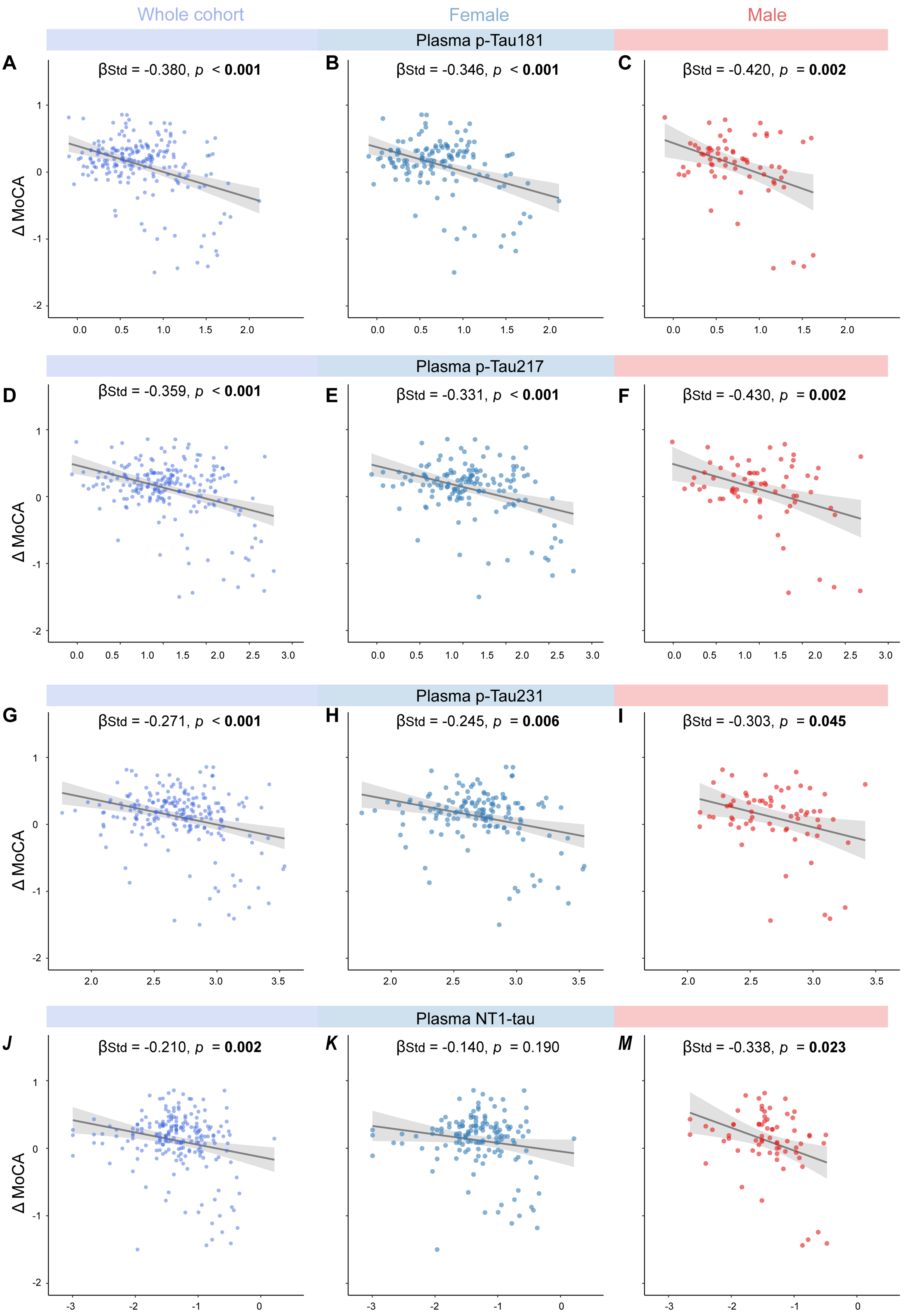
Whole cohort
Female
Male
Plasma p-Tau181
A
βStd = -0.380, p < 0.001
B
βStd = -0.346, p < 0.001
C
βStd = -0.420, p = 0.002
Δ MoCA
Plasma p-Tau217
D
βStd = -0.359, p < 0.001
E
βStd = -0.331, p < 0.001
F
βStd = -0.430, p = 0.002
Δ MoCA
Plasma p-Tau231
G
βStd = -0.271, p < 0.001
H
βStd = -0.245, p = 0.006
I
βStd = -0.303, p = 0.045
Δ MoCA
Plasma NT1-tau
J
βStd = -0.210, p = 0.002
K
βStd = -0.140, p = 0.190
M
βStd = -0.338, p = 0.023
Δ MoCA

Supplementary Figure 6. Associations of longitudinal cognitive decline with plasma tau biomarkers. Association of Δ MoCA score with plasma p-tau181 (A-C), p-tau217 (D-F), p-tau231 (G-I), and NT1-tau (J-M) in different subgroups (whole, female, male). Notably, the standardized regression coefficients (β_{Std}) and values were computed using a generalized linear model, adjusting for age and APOE-ε4. p-Tau = phosphorylated tau; NT1-tau = N-terminal tau.
